# Determination of Odor Intensity of Binary Gas Mixtures Using Perceptual Models and an Electronic Nose Combined with Fuzzy Logic

**DOI:** 10.3390/s19163473

**Published:** 2019-08-08

**Authors:** Bartosz Szulczyński, Jacek Gębicki

**Affiliations:** Department of Process Engineering and Chemical Technology, Chemical Faculty, Gdansk University of Technology, 11/12 G. Narutowicza Str., 80-233 Gdańsk, Poland

**Keywords:** electronic nose, fuzzy logic, odor intensity, odor interaction, gas sensors, perceptual model, odors

## Abstract

Measurement and monitoring of air quality in terms of odor nuisance is an important problem. From a practical point of view, it would be most valuable to directly link the odor intensity with the results of analytical air monitoring. Such a solution is offered by electronic noses, which thanks to the possibility of holistic analysis of the gas sample, allow estimation of the odor intensity of the gas mixture. The biggest problem is the occurrence of odor interactions between the mixture components. For this reason, methods that can take into account the interaction between components of the mixture are used to analyze data from the e-nose. In the presented study, the fuzzy logic algorithm was proposed for determination of odor intensity of binary mixtures of eight odorants: *n*-Hexane, cyclohexane, toluene, *o*-xylene, trimethylamine, triethylamine, α-pinene, and β-pinene. The proposed algorithm was compared with four theoretical perceptual models: Euclidean additivity, vectorial additivity, U model, and UPL model.

## 1. Introduction

The scent, which is a sensory impression, is relatively difficult to quantify. In research on odorous compounds and in attempts to describe it, four basic fragrance characteristics are taken into account: The odor concentration, odor intensity, hedonic tone, and odor threshold [1,2,3,4]. The odor intensity (OI) depends on the number of fragrance molecules that contact the olfactory receptors, i.e., on its concentration in the inhaled air. The odor intensity is defined as the “odor strength” that will be triggered by a specific fragrance stimulus. In the case of gas mixtures whose components are odor compounds, there is a discrepancy between the perceived smell and the total scent (which is the sum of the fragrances of the individual components). This is caused by the occurrence of the odor interaction, based on the mutual masking, synergy or the inhibition phenomenon [5]. Studies on the types of olfactory interactions have been conducted for a very long time, but so far have not led to the explanation of the mechanism of these processes. The objects of experimental research are usually air samples containing only two or three types of odorants [6,7,8,9].

The relationship between physical stimuli acting on the senses and mental feelings is dealt with in the field called psychophysics. In the case of olfactory interactions, the models of the odor interaction are considered, which describe the dependence of the odor intensity of air containing mixtures of impurities from:

- The odor intensity that would cause components of the mixture if they were present individually (perceptual models);
- concentrations of components of the mixture and their psychophysical characteristics (psychophysical models).

None of the numerous models developed represent a general model. Therefore, the problem of predicting the odor intensity of gaseous mixtures has not been successfully solved, mainly due to the occurrence of interactions between fragrances of the mixture, causing mutual enhancement or weakening of the scent.

In the 70s and 80s of the twentieth century, considerable attention was given to developing mathematical models for predicting the intensity and quality of odor mixtures. Several mathematical models were proposed to estimate the odor intensities of mixtures, as they are perceived by humans: Euclidean additivity, vectorial model, U model or UPL model [10,11,12,13,14]. However, the use of these models requires prior sensory measurements that are expensive and time-consuming. 

In recent years, there has been a lot of interest in the subject of the instrumentation of odor measurement using devices called electronic noses [15,16,17,18]. Electronic noses are the analytical devices, which in their functioning resemble the human sense of smell [19,20,21,22,23,24,25]. Sensors are the analogs of the olfactory receptors. They turn the chemical information into an analytically useful signal. Then, the signal is sent to the recognition system, which in the case of the human body, is the brain, and in the case of the e-nose, is the appropriate mathematical algorithm [26]. The most commonly used data processing methods are: Principal component analysis (PCA), principal component regression (PCR), partial least square regression (PLSR), fuzzy logic (FL), and artificial neural networks (ANN) [27,28,29,30,31,32,33,34,35].

Instrumentation of odor measurement will allow the use of instrumental methods wherever the measurement, using the human sense of smell, will be impossible or even dangerous. In addition, the use of electronic noses will significantly reduce the time and costs of a single analysis and will enable continuous monitoring systems. Odor measurement instrumentalization is possible because the mutual relations between the sensor signals may correspond to odor interactions in the mixture.

In the presented studies, four theoretical perceptual models were compared to those obtained using the electronic nose, in which fuzzy logic was used as the method of analyzing measurement data. The research was conducted with the use of eight odorous compounds: *n*-Hexane, cyclohexane, toluene, *o*-xylene, trimethylamine, triethylamine, α-pinene, and β-pinene. The coefficient of odor interaction for the binary mixtures of the abovementioned compounds was determined. The mixtures were generated using a developed gas mixture generator. In the research, a prototype of an electronic nose equipped with eight gas chemical sensors (one photoionization, two electrochemical, and five metal oxide semiconductor sensors) was used.

## 2. Materials and Methods

### 2.1. Gas Mixture Generator

**Figure 1:**
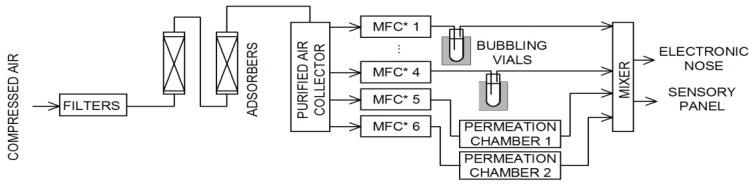
Gas mixture generator schematic (MFC—mass flow controllers).

All samples (the single substance in air and binary mixtures) were prepared using a gas mixture generator prototype. The device operates based on two methods of obtaining standard gas mixtures: The bubbling system and permeation tubes. The device operation diagram is presented in Figure 1. 

The compressed air was initially cleaned by a set of filters and adsorbers and then stored in a stainless steel collector. Using mass flow controllers (red-y series, Vögtlin Instruments GmbH, Aesch, Switzerland), the air was directed to bubbling vials or permeation chambers (in which the permeation tubes were placed). The temperature of all device modules was precisely controlled and regulated. The last part of the generator is a mixer. This module allowed mixing all streams, as well as due to the additional airline, it was possible to dilute the sample to obtain the desired concentration of the components of the mixture. The output of the device was adapted to: Take a sample into a gas-tight syringe (for gas chromatography analysis), insert the sample into the electronic nose sensor chamber, and present the sample to the members of the sensory panel. For proper operation of the gas mixture generator, it was calibrated using the gas chromatography technique (GC). Randomly selected generated samples were also analyzed using GC to ensure correct concentration values.

The concentrations obtained using the bubbling system (Equations (1) and (2)) and using self-manufactured permeation tubes (Equation (3)) can be calculated using the following formulas:

(1) $$\;W=\frac{P^{0}\cdot M\cdot \dot{V}}{10^{3}\cdot R\cdot T}$$

(2) $$c=\frac{24.04\cdot W}{10^{-6}\cdot \dot{V}\cdot M}$$

(3) $$c=\frac{E}{\rho \cdot \dot{V}}$$

where: *c*—concentration of the substances in a stream of a carrier gas [ppm], *W*—the mass flow of the evaporated substance [mg s^−1^], *P^0^*—the vapor pressure at the given temperature [Pa], *M—*the molar mass of the evaporated substance [g mol^−1^], $\dot{V}$—the volumetric flow rate of the stream of the carrier gas [mL s^−1^], *R*—the gas constant [J mol^−1^ K^−1^], *E*—the permeation ratio [ng s^−1^], *ρ*—the density of the gas component subject to the process of permeation [ng nL^−1^].

### 2.2. Odorants

Eight chemical substances were used in the presented research. They represented four groups of odorant compounds: The alkanes and cycloalkanes, aromatic hydrocarbons, amines, and terpenes. Their basic properties are presented in Table 1.

### 2.3. Sensory Analysis

Twenty-five volunteers participated in preliminary investigations, which utilized an air mixture of n-butanol prepared at 5 concentrations: 0, 10, 20, 40, 80 ppm. During two days, each volunteer carried out ten analyses aimed at the identification of an individual perceptibility threshold with respect to the n-butanol solutions. The preliminary investigations allowed the selection of volunteers, who fulfilled the criterion of individual repeatability required:

(4) $$10^{s}\leq 2.3$$

where: s—standard deviation of the individual odor evaluations.

The volunteers (5 women and 5 men) aged 22–35 were selected to participate in the sensory analysis. They were trained for one week before the tests. The volunteers were non-smokers and their physical, as well as their mental condition, was evaluated as very good. They did not eat or drink for an hour before the test in order to avoid interference from foreign odors with the aroma substances under examination. The task for panelists was to determine the odor intensity (OI) of the inhaled sample using the German standard VDI 3940 scale (Table 2). 

### 2.4. Stevens’ and Weber–Fechner Laws Coefficients Determination

The dependence of the odor intensity on the concentration of a single substance can be described using the Weber–Fechner (Equation (5)) and Stevens’ (Equation (6)) laws:

(5) $$\mathrm{OI}=k_{WF}\cdot \log\frac{C}{C_{OT}}$$

(6) $$\mathrm{OI}=k_{S}\cdot C^{n}$$

where: OI—odor intensity; k_WF_, k_s_, n—experimentally determined coefficients, C—odorant concentration, and C_OT_—odorant odor threshold.

Sensory analysis of five concentration levels of each odorant was performed. Each concentration was two-fold higher than the preceding. For the obtained results, two plots were performed: *OI = f(logC)* for the Weber–Fechner coefficient and odor threshold determination and *log OI = f(logC)* for the Stevens’ law coefficients determination for each odorant.

### 2.5. Theoretical Prediction of Odor Intensity of Binary Mixtures 

For theoretical prediction of the odor intensity of the prepared binary mixtures of odorants, four theoretical models were used: The vectorial model, Euclidean additivity, U model, and UPL model.

#### 2.5.1. Vectorial Model

The form of the model was proposed by Berglund in 1973 [10]. The formula of olfactory interaction in the binary mixture (A and B) is presented in Equation (7):

(7) $${\mathrm{OI}}_{\mathrm{AB}}=\sqrt{{\mathrm{OI}}_{A}^{2}+{\mathrm{OI}}_{B}^{2}+2\cdot {\mathrm{OI}}_{A}\cdot {\mathrm{OI}}_{B}\cdot \cos\alpha_{\mathrm{AB}}}$$

where cosα_AB_ is the interaction coefficient between odorant A and odorant B. For proper use, it is necessary to experimentally determine the interaction coefficient value using Equation (8):

(8) $$\cos\alpha_{\mathrm{AB}}=\frac{{\mathrm{OI}}_{\mathrm{AB}}^{2}-{\mathrm{OI}}_{A}^{2}-{\mathrm{OI}}_{B}^{2}}{2\cdot {\mathrm{OI}}_{A}\cdot {\mathrm{OI}}_{B}}$$

#### 2.5.2. Euclidean Additivity Model

The Euclidean additivity model is a particular case of the vectorial model, where it is assumed that there are no mutual interactions between the components of the mixture (cosα_AB_ = 0):

(9) $${\mathrm{OI}}_{\mathrm{AB}}=\sqrt{{\mathrm{OI}}_{A}^{2}+{\mathrm{OI}}_{B}^{2}}$$

In the presented research, this model was used as the reference model for the mutual comparison of the obtained results.

#### 2.5.3. U Model

Patte and Laffort proposed the U model for binary mixtures in 1979 [37]. It is based on Equation (10):

(10) $$OI_{\mathrm{AB}}=OI_{A}+OI_{B}+2\cdot \cos\alpha_{\mathrm{AB}}\cdot \sqrt{OI_{A}\cdot OI_{B}}$$

As in the case of the vectorial model, the interaction coefficient must be determined experimentally by using the Equation (11):

(11) $$\cos\alpha_{\mathrm{AB}}=\frac{{\mathrm{OI}}_{\mathrm{AB}}-{\mathrm{OI}}_{A}-{\mathrm{OI}}_{B}}{2\cdot \sqrt{{\mathrm{OI}}_{A}\cdot {\mathrm{OI}}_{B}}}$$

#### 2.5.4. UPL Model

The UPL model is the modification of U model (Equation (10)) proposed in 1982 by Laffort and Dravnieks [13]. The modification includes the interaction coefficient. In this case, cosα_AB_ reflects only the Stevens’ power law determined for a single component. The first step for determination of the interaction coefficient in a binary mixture is to determine the coefficient for each single component using Equation (12):

(12) $$\cos\alpha_{A}=2^{n_{A}-1}-1$$

Equation (12) is strictly correct only when OI_A_ = OI_B_. In the next step, it is possible to determine the interaction coefficient between the mixture components:

(13) $$\cos\alpha_{\mathrm{AB}}=\frac{\cos\alpha_{A}\cdot OI_{A}+\cos\alpha_{B}\cdot OI_{B}}{OI_{A}+OI_{B}}$$

### 2.6. Electronic Nose Analysis 

In the presented research, the analyses were carried out using an electronic prototype equipped with a measuring chamber containing eight sensors (Table 3). 

**Figure 2:**
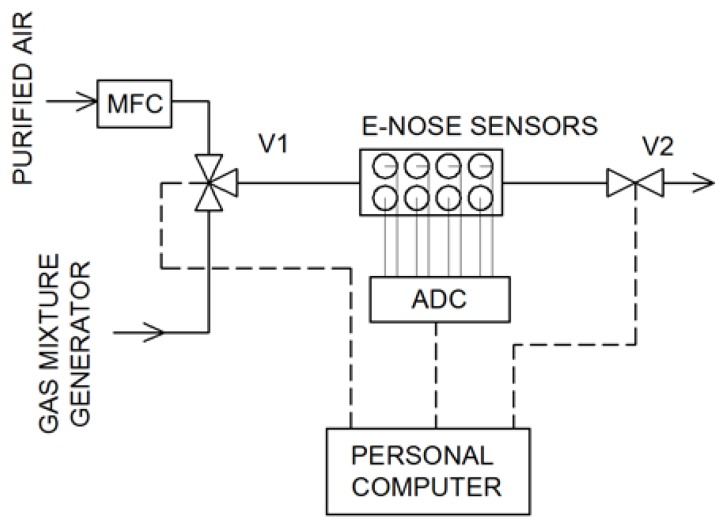
Electronic nose experimental setup (MFC—mass flow controller, ADC—analog-to-digital converter).

The schematic of the measurement system is presented in Figure 2. Purified air flowed through the system at a constant flow rate of 300 cm^3^ min^−1^. It was controlled by a mass flow controller. By changing the position of the valve V1, the sample from the gas mixture generator flowed through the measurement chamber. The electronic nose worked in the stop-flow mode [38]: The sample flow time was 40 s and the stop time of the mixture in the sensors chamber was—20 s (after closing the V2 valve). After this time, the purified air was returned to the measurement chamber for the regeneration of the sensors. Signals from the sensors were recorded using an 8-chanel 12-bit analog-to-digital converter and saved on the computer. The data analysis and other calculations were performed in RStudio Desktop (v. 1.1.463) software [39] using R [40].

**Figure 3:**
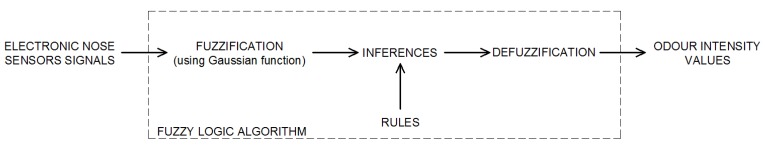
Fuzzy logic algorithm for the odor intensity estimation using electronic nose sensors signals.

One of the most interesting approaches in the field of e-nose data analysis is fuzzy logic. The classical logic system is based on the two values, mostly represented by 0 and 1, or *true* and *false*. The boundary between them is defined and unchanging. Fuzzy logic is an extension of the classical approach to approach closer to the human brain; it introduces additional values between standard *true* and *false*. Blurring the boundaries between them gives the opportunity to come up with values between this interval (e.g., *almost false*, *half truth*). The proposed scheme of using fuzzy logic to estimate the odor intensity is presented in Figure 3 and described in previous research [41,42]. 

**Figure 4:**
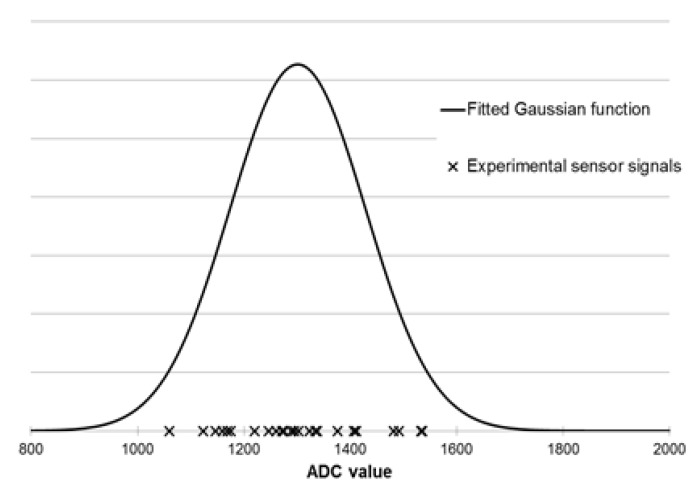
Gaussian membership function determined for TGS2603 sensor based on measurements of sample with odor intensity equal to 3 (distinct odor).

In this work, Gaussian membership functions were used. The defining of fuzzy sets for each sensor is presented in Figure 4. For each sensor at every odor intensity level (Table 2), all signal distributions were determined using the Gaussian function (using the mean and standard deviation values). In the next step, based on the measurements results, a set of rules were developed. An example of the rule is presented in Equation (14):

*IF(S_1_∈ Very weak) AND...AND(S_8_ ∈ Very weak) THEN (OI ∈ Very weak)* (14)

The proposed fuzzy logic algorithm proceeds in three stages (Figure 3). At the input of the model, eight input variables (each sensor signals) were introduced. In the fuzzification block, the degree of belonging of the individual values to the fuzzy sets was calculated. In the next stage, using the created rules, the resulting function of the model output was calculated. At the defuzzification stage, the resulting affinity function was the basis for calculating the value of the sample odor intensity (output variable for the fuzzy logic algorithm). In the presented research, the center of the gravity mechanism was used for this purpose. 

## 3. Results

After the performance of sensory analysis of single-component samples at five concentration levels, the values of Weber–Fechner and Stevens’ power law coefficients were calculated. The results are presented in Table 4.

Using determined Weber–Fechner law formulas, for each odorant, the concentrations corresponding to odor intensity values equal to 1, 2 and 3 were calculated. The values are shown in Table 5.

In the next step of the research, 56 binary mixtures were generated. A total of 28 mixtures were generated in such a way that the concentrations of the individual substances were equal to an odor intensity equal to 1 (e.g., 1.1 ppm of toluene and 1.5 ppm of *o*-xylene) and 28 mixtures corresponding to odor intensity equal to 2 (e.g., 1.5 ppm trimethylamine and 10 ppm α-pinene). The odor intensity of the mixtures was evaluated using sensory analysis in triplicate for each sample. In this way, the mean value of the sensory odor intensity for each sample was determined, which was then used to determine the odor interaction coefficients according to Equations (8, 11–13). The results for each theoretical calculation are presented in Table 6, Table 7 and Table 8.

**Figure 5:**
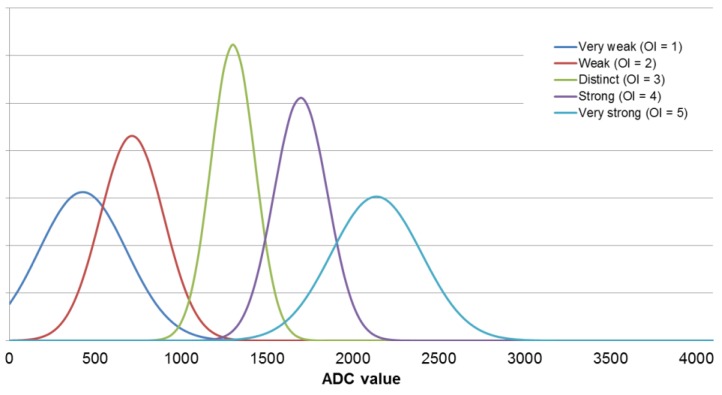
Gaussian membership functions calculated for the TGS2603 sensor.

The electronic nose—fuzzy logic system was developed using the measurement results obtained for the e-nose analysis of single component samples at odor intensity levels from 1 to 5. Using all results for each sensor, its maximum signal value distribution was determined using the Gaussian function. The mean values and standard deviations were calculated and transferred into membership functions. An exemplary fuzzification step for the TGS2603 sensor is shown in the Figure 5. 

**Figure 6:**
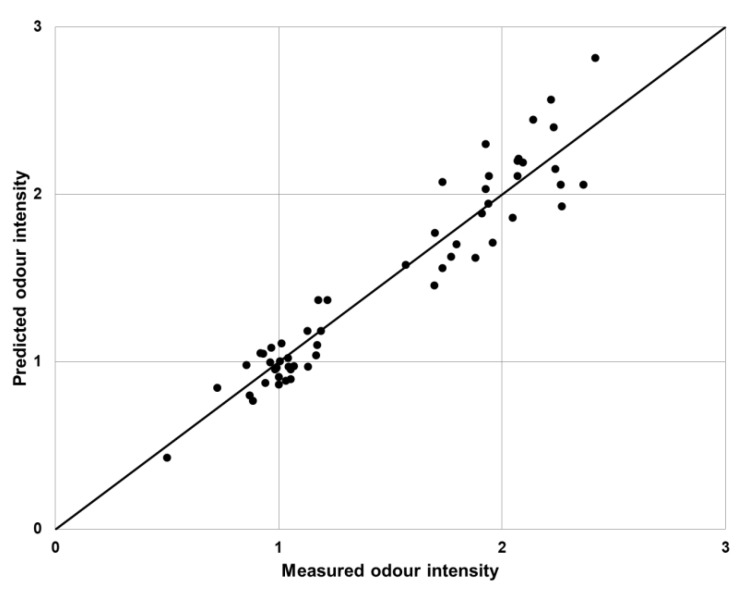
Proposed fuzzy logic algorithm validation plot.

Validation of the proposed algorithm was performed using a measured-predicted plot (Figure 6). The measured values were obtained using a sensory analysis and the predicted values were the results of the e-nose analysis (determined using the developed fuzzy logic algorithm). 

**Figure 7:**
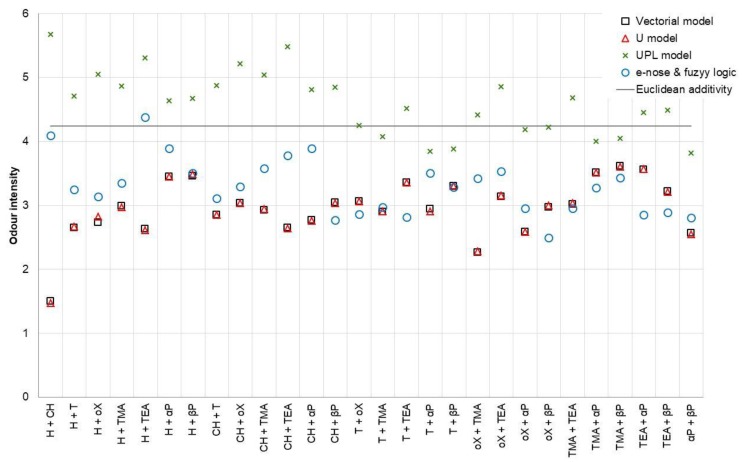
Odor intensity prediction models comparison (H—*n*-hexane, CH—cyclohexane, T—toluene, oX—*o*-xylene, TMA—trimethylamine, TEA—triethylamine, αP—α-pinene, βP—β-pinene).

For comparison of the theoretical models, sensory analysis, and values obtained using the electronic nose and fuzzy logic, 28 binary mixtures were generated in such a way that the concentrations of the individual substances were equal to an odor intensity equal to 3 (e.g., 74.1 ppm α-pinene and 28.5 ppm β-pinene). The mixtures were investigated using the sensory panel and the electronic nose. Three replicates were made for each sample. The theoretical values were determined using the vectorial model (Equation (7)), Euclidean additivity model (Equation (9)), U model (Equation (10)), and UPL model. As an interaction factor, the mean values from Table 6; Table 7 were used. The comparison of the obtained is shown in Figure 7. 

For quantitative comparison of the perceptual models and the fuzzy logic algorithm, the mean squared prediction error (*MSPE_PM_*) was used:

(15) $$MSPE_{PM}=\frac{\sum {\left(OI_{PM}-OI_{FL}\right)}^{2}}{n}$$

where: *OI_PM_*: odor intensity determined using one of the perceptual models, *OI_FL_*_:_ odor intensity of the same sample determined using fuzzy logic algorithm, *n*: number of samples. The *MSPE_PM_* are presented in Table 9.

## 4. Discussion

In the presented studies, the odor interaction coefficients were determined for three theoretical models (the vectorial additivity model, U model, and UPL model) used to determine the odor intensity of the binary mixtures of based on the intensity of individual components. The research was carried out using eight odorants, belonging to five groups of chemical compounds. Considering the results presented in Table 6, Table 7 and Table 8, it should be stated that all determined coefficients were negative and mostly had values between −0.25 and −0.60. For the vectorial and U models, the lowest values were observed for the interaction between n-hexane and cyclohexane, which indicated the occurrence of mutual inhibition of the odor intensity. However, the highest values appeared mostly in mixtures in which one of the components was α-pinene or β-pinene. This phenomenon may have been caused by the positive hedonic tone of these substances scents. 

In the case of the vectorial additivity and U model, it was possible to compare the interaction coefficients determined for mixtures generated at two odor intensity levels: 1 and 2. In both cases, these values were very similar to each other, which allowed stating that at low odor intensity levels, these coefficients are unchanged for a given pair of compounds. 

The interaction coefficients values obtained for the UPL model differed significantly from the other two models. It is connected with the theoretical determination of coefficients in this model, that when determining them, there was no feedback with the values obtained by the sensory panelist team. Analyzing the results presented in Figure 7, it can be seen that the partial compliance of the UPL model with the others only occurred for mixtures containing α-pinene or β-pinene. In all cases, this model overestimated the odor intensity values. 

The use of an electronic nose combined with the proposed fuzzy logic algorithm gave satisfactory results, which in most mixtures, was similar to the vectorial and U models (mean squared prediction error equal to 0.54 and 0.53, respectively). The worst fit was presented by the UPL model, where *MSPE_PM_* was equal to 3.77. Discrepancies between the values occurred for mixtures containing trimethylamine or triethylamine. This was caused by the very low odor thresholds of these substances. At low concentrations of these substances, their scent could be felt as strong, while the substance was not detected by sensors installed in the electronic nose. The proposed method of data analysis, based on fuzzy logic, very well reflected the sensory panel feelings, especially for low values of the odor intensity (Figure 6). However, the results presented only show a reference to the determination of the odor intensity of binary mixtures, which are very rare in real conditions.

When treating the Euclidean additivity model as a simple reference model, it should be pointed out that in all cases, we are dealing with the attenuation of the intensity in relation to the simple Euclidean summation. This clearly proves the existence of mutual interactions between components in the binary odor mixtures. 

The obtained results indicated a similar dependence, as in the case of using other models. Yan et al. [43] proposed a model of odor interactions for binary mixtures of benzene and its derivatives, employing a partial differential equation (PDE), which was compared with the U model, strongest component model, and additivity model. In other studies, Yan et al. [14] proposed a modified vector model and checked its use to study the interaction in binary, ternary, and quaternary mixtures. As in the case of the presented research, he obtained good agreement between the predicted OI values with those measured for the binary mixtures. Chen [44] compared the U model and modified vector model for benzene, ethylbenzene, and toluene binary mixtures. In most of the results, there was an odor intensity synergy effect for the studied mixtures. The proposed application of the electronic nose along with the fuzzy logic algorithm allows continuous measurements, which in the case of the other presented solutions, is possible only with the use of the PDE model, which requires more computing resources.

## 5. Conclusions

In the presented study, four theoretical perceptual models were compared to those obtained using the electronic nose, in which fuzzy logic was used as the method of analyzing measurement data. The analysis of the obtained results allowed us to conclude that the use of an electronic nose as an instrumental tool for assessing the odor of binary gas mixtures is fully justified and purposeful. However, the use of fuzzy logic introduces the need to properly select of the membership function, defuzzification mechanism, and set of rules, which requires some expert knowledge.

With more complex mixtures, the use of an electronic nose can be problematic, mainly due to the occurrence of mutual odor interactions between the mixture components. Solving the problem will certainly help the development of sensory techniques associated with constructing more sensitive, specific, and selective sensors with lower limits of detection. Another approach is the use of more sophisticated methods of data analysis, which allow the evaluation of the interaction of fragrances by analysis of signals obtained from e-nose sensors. In this field, artificial neural networks (ANN) are the most valuable methods for sensor data processing. This is related to their similarity to the functioning of the human brain, which is the most important part of the human sense of smell. However, the methods of creating an optimal neural network are much more complicated and time-consuming compared to fuzzy logic, mainly due to the need to determine the number of layers, the number of neurons in each layer of the network and the type of activation function.

Mutual comparison of the perceptual theoretical models has allowed us to demonstrate the usefulness of these models, based on the interaction coefficients determined using sensory analysis (i.e., the vectorial model and U model). The UPL model only takes into consideration the power law exponents of the individual components. This means that the evaluation of the interaction between the two components of the mixture using the UPL model is in most cases incorrect. 

## Figures and Tables

**Table 1:** Basic properties of odorants sing in the research.

Group of Odorants	Odorant	Molecular Mass [g mol^−1^]	Density ^1^ [g cm^−3^]	Vapor Pressure ^1^ [Pa]	Odor Type	Odor Threshold in Air ^2^ [ppm]
alkanes & cycloalkanes	*n*-hexane	86.178	0.6606	17,600	petrolic	1.5
	cyclohexane	84.162	0.7781	10,400	sweet, gasoline-like	2.5
aromatic hydrocarbons	Toluene	92.141	0.87	2800	sweet, pungent, benzene-like	0.33
	*o*-xylene	106.168	0.88	933	sweet	0.38
amines	trimethylamine	59.112	0.627	188,700	fishy, ammoniacal	0.000032
	triethylamine	101.193	0.7255	8506	fishy, pungent	0.0054
terpenes	α-pinene	136.238	0.858	536	pine, resinous	0.018
	β-pinene	136.238	0.872	391	woody-green pine-like	0.033

^1^ values determined at 20.0 °C, ^2^ according to Reference [36].

**Table 2:** German standard VDI 3940 odor intensity scale.

Intensity Level	Odor Strength
0	Not perceptible
1	Very weak
2	Weak
3	Distinct
4	Strong
5	Very strong
6	Extremely strong

**Table 3:** Types of chemical sensors used in electronic nose prototype.

Sensor Type	Model	Manufacturer	Detected Compounds
Photoionization	MiniPID	Ion Science	Aromatic hydrocarbons, VOCs
Electrochemical	FECS44-100	Figaro	ammonia
Electrochemical	FECS50-100	Figaro	Hydrogen sulfide
Metal Oxide Semiconductor	TGS2600	Figaro	Air contaminants
Metal Oxide Semiconductor	TGS2602	Figaro	VOCs and odorous gases
Metal Oxide Semiconductor	TGS2603	Figaro	Air contaminants (triethylamine, mercaptanes, etc.)
Metal Oxide Semiconductor	TGS823	Figaro	Organic solvent vapors
Metal Oxide Semiconductor	TGS8100	Figaro	Air contaminants

**Table 4:** Weber–Fechner and Stevens’ law coefficients calculated based on experimental measurements.

Odorant	Weber–Fechner Law		Stevens’ Power Law	
	*k_WF_*	*C_OT_* [ppm]	*n*	*k_S_*
*n*-hexane	2.14 ± 0.23	1.1	0.874 ± 0.061	0.255
cyclohexane	1.93 ± 0.22	1.1	0.961 ± 0.041	0.191
toluene	1.96 ± 0.29	0.34	0.382 ± 0.125	1.119
*o*-xylene	2.23 ± 0.25	0.53	0.614 ± 0.115	0.710
trimethylamine	2.14 ± 0.21	0.17	0.496 ± 0.104	1.437
triethylamine	2.39 ± 0.26	0.35	0.771 ± 0.145	0.821
α-pinene	1.15 ± 0.19	0.18	0.330 ± 0.024	0.927
β-pinene	1.45 ± 0.20	0.24	0.361 ± 0.025	1.004

**Table 5:** Concentration of individual substances used for generation mixture characterized with specific odor intensity value.

Odorant	Concentration [ppm]		
	*OI* = 1	*OI* = 2	*OI* = 3
*n*-hexane	3.2	9.3	27.1
cyclohexane	3.7	12.1	39.7
toluene	1.1	3.6	11.5
*o*-xylene	1.5	4.2	11.6
trimethylamine	0.5	1.5	4.4
triethylamine	0.9	2.4	6.4
α-pinene	1.3	10	74.1
β-pinene	1.2	5.8	28.5

**Table 6:** Vectorial additivity odor interaction coefficients.

	Odor Intensity = 1								Odor Intensity = 2							
	Hexane	Cyclohexane	Toluene	*o*-xylene	TMA ^1^	TEA ^2^	α-pinene	β-pinene	Hexane	Cyclohexane	Toluene	*o*-xylene	TMA ^1^	TEA ^2^	α-pinene	β-pinene
hexane	-								-							
cyclohexane	−0.88	-							−0.87	-						
toluene	−0.60	−0.56	-						−0.62	−0.54	-					
*o*-xylene	−0.55	−0.48	−0.48	-					−0.62	−0.50	−0.48	-				
TMA	−0.51	−0.54	−0.54	−0.73	-				−0.5	−0.51	−0.53	−0.70	-			
TEA	−0.61	−0.61	−0.38	−0.44	−0.50	-			−0.62	−0.61	−0.37	−0.47	−0.49	-		
α -pinene	−0.33	−0.57	−0.51	−0.63	−0.31	−0.29	-		−0.35	−0.58	−0.53	−0.63	−0.32	−0.30	-	
β-pinene	−0.33	−0.48	−0.39	−0.51	−0.27	−0.41	−0.63	-	−0.34	−0.49	−0.4	−0.51	−0.28	−0.44	−0.64	-

^1^ TMA—trimethylamine, ^2^ TEA—triethylamine.

**Table 7:** U model odor interaction coefficients.

	Odor Intensity = 1								Odor Intensity = 2							
	Hexane	Cyclohexane	Toluene	*o*-xylene	TMA ^1^	TEA ^2^	α-pinene	β-pinene	Hexane	Cyclohexane	Toluene	*o*-xylene	TMA ^1^	TEA ^2^	α-pinene	β-pinene
hexane	-								-							
cyclohexane	−0.76	-							−0.75	-						
toluene	−0.55	−0.53	-						−0.56	−0.52	-					
*o*-xylene	−0.52	−0.49	−0.49	-					−0.54	−0.50	−0.49	-				
TMA	−0.51	−0.52	−0.52	−0.63	-				−0.50	−0.50	−0.51	−0.61	-			
TEA	−0.56	−0.56	−0.44	−0.47	−0.50	-			−0.57	−0.56	−0.44	−0.48	−0.49	-		
α-pinene	−0.42	−0.54	−0.51	−0.57	−0.41	−0.40	-		−0.43	−0.54	−0.52	−0.57	−0.42	−0.41	-	
β-pinene	−0.42	−0.49	−0.45	−0.50	−0.4	−0.46	−0.57	-	−0.42	−0.50	−0.45	−0.50	−0.40	−0.47	−0.58	-

^1^ TMA—trimethylamine, ^2^ TEA—triethylamine.

**Table 8:** UPL model odor interaction coefficients.

	Hexane	Cyclohexane	Toluene	*o*-xylene	TMA ^1^	TEA ^2^	α-pinene	β-pinene
hexane	-							
cyclohexane	−0.06	-						
toluene	−0.21	−0.19	-					
*o*-xylene	−0.16	−0.13	−0.29	-				
TMA	−0.19	−0.16	−0.32	−0.26	-			
TEA	−0.11	−0.09	−0.25	−0.19	−0.22	-		
α-pinene	−0.23	−0.20	−0.36	−0.30	−0.33	−0.26	-	
β-pinene	−0.22	−0.19	−0.35	−0.30	−0.33	−0.25	−0.36	-

^1^ TMA—trimethylamine, ^2^ TEA—triethylamine.

**Table 9:** Mean squared prediction error values determined for perceptual models.

	Vectorial Model	U Model	Euclidean Additivity	UPL Model
*MSPE_PM_*	0.54	0.53	1.09	3.77
